# The Combination of Chitosan-Based Biomaterial and Cellular Therapy for Successful Treatment of Diabetic Foot—Pilot Study

**DOI:** 10.3390/ijms25158388

**Published:** 2024-08-01

**Authors:** Filip Humenik, Katarína Vdoviaková, Lenka Krešáková, Ján Danko, Mária Giretová, Ľubomír Medvecký, Peter Lengyel, Ján Babík

**Affiliations:** 1 Department of Morphological Sciences, University of Veterinary Medicine and Pharmacy in Košice, 041 81 Košice, Slovakia; katarina.vdoviakova@uvlf.sk (K.V.); lenka.kresakova@uvlf.sk (L.K.); jan.danko@uvlf.sk (J.D.); 2 Division of Functional and Hybrid Systems, Institute of Materials Research of SAS, 040 01 Košice, Slovakia; mgiretova@saske.sk (M.G.); lmedvecky@saske.sk (Ľ.M.); 3 Clinic of Burns and Reconstructive Medicine, AGEL Hospital, 040 15 Košice-Šaca, Slovakia; peter.lengyel@nke.agel.sk (P.L.); jan.babik@nke.agel.sk (J.B.)

**Keywords:** mesenchymal stem cells, fibroblasts, chitosan-based biomaterial, diabetic foot, wound

## Abstract

Diabetic foot ulceration is one of the most common complications in patients treated for diabetes mellitus. The presented pilot study describes the successful treatment of diabetic ulceration of the heel with ongoing osteomyelitis in a 39-year-old patient after using a combination of modified chitosan-based biomaterial in combination with autologous mesenchymal stem cells isolated from bone marrow and dermal fibroblasts. The isolated population of bone marrow mesenchymal stem cells fulfilled all of the attributes given by the International Society for Stem Cell Research, such as fibroblast-like morphology, the high expression of positive surface markers (CD29: 99.1 ± 0.4%; CD44: 99.8 ± 0.2% and CD90: 98.0 ± 0.6%) and the ability to undergo multilineage differentiation. Likewise, the population of dermal fibroblasts showed high positivity for the widely accepted markers collagen I, collagen III and vimentin, which was confirmed by immunocytochemical staining. Moreover, we were able to describe newly formed blood vessels shown by angio CT and almost complete closure of the skin defect after 8 months of the treatment.

## 1. Introduction

Diabetic foot syndrome, with its complications, is a primary cause of hospitalization for patients with diabetes. It significantly contributes to the increase in morbidity and mortality of these patients and is the most common non-traumatic cause of limb amputation in the USA and Europe. It is estimated that patients with diabetes mellitus have an approximate 15% risk of developing foot ulceration during their lifetime, and the prevalence of ulceration ranges from 4 to 10% [1]. The price of is also not insignificant, with the treatment of patients with developed diabetic foot syndrome rising to several billion dollars in the US annually [2]. Diabetic foot is defined by the WHO as a foot of a patient with diabetes that is neuropathic, vascular and articular and that, as a result damage, is chronically at risk of infection, ulceration, gangrene and destruction of deep tissue structures, ultimately resulting in the amputation of limbs [3]. Diabetic foot syndrome complications are always a serious threat to the patient, not only with regard to the limb, but also to the patient’s overall state of health. By improving the treatment and nursing care of diabetic foot lesions, it is possible to reduce the number of limb amputations. Although not every diabetic foot ulceration can be prevented, with the help of well-documented preventive measures in the framework of cooperation between the doctor and the patient, it is realistic to significantly reduce the incidence of diabetic foot syndrome. As early as the time of diagnosis, approximately 10% of diabetics have angiopathy or neuropathy and 20–30% have nephropathy or retinopathy [4,5,6]. The gold standard of diabetic foot management and therapy includes debridement of the wound, management of any infection, revascularization procedures when indicated, and off-loading of the ulcer [7,8]. Other methods have also been suggested to be beneficial as add-on therapies, such as hyperbaric oxygen therapy, use of advanced wound care products, and negative-pressure wound therapy (NPWT) [9]. However, it is not always possible to achieve satisfactory results after using the above procedure. In such cases, the next step is the use of alternative methods, such as the application of stem cells, fibroblasts, growth factors, biomaterials or perinatal derivatives such as amnion/chorion membrane [10,11,12,13]. Fibroblasts play an important role in the wound healing process, primarily by fibrin clot lysis, extracellular matrix formation, collagen formation and wound contraction [14]. Similarly, MSCs play an important role in the wound-healing process. They participate in the homeostasis phase by the promotion of coagulation due to the high tissue factor content and phosphatidylserine [15]. MSCs modify the inflammatory phase via the production of bioactive molecules that polarize M1 pro-inflammatory macrophages to the M2 anti-inflammatory type or suppress the effect of NK cells. They achieve this via their capacity to downregulate TNF-α-dependent inflammation and by triggering the TGF-β1 production that has a key role in wound healing and inflammation control [16,17]. The proliferative phase of healing is characterized via the widespread activation of fibroblasts, keratinocytes, endothelial cells and macrophages to orchestrate wound closure, angiogenesis and matrix deposition. Similarly, MSCs can improve the proliferative phase of wound healing [18]. The mechanism of influencing the proliferative phase of wound healing is primarily based on the production of growth factors such as VEGF, bFGF, KFG, EGF, which ae closely related to granulation and epithelization [19,20,21]. The maturation phase is known as the last step of the wound repair process. MSCs attend to this phase by the production of numerous soluble factors and cytokines that suppress myofibroblast differentiation, enhance epithelial–mesenchymal transition, or show anti-fibrotic and proangiogenic potential (hepatocyte growth factor, prostaglandin E2, IL-10 adrenomedulin, vein endothelial growth factor, epidermal growth factor or CXCL2) [22,23]. Another approach to the therapy of the diabetic foot is based on the use of polymeric materials, in the form of membranes, films, hydrogels, foam or bandages [24]. Of the mentioned biomaterials, chitosan-based biomaterials have been widely researched and used in the regenerative medicine of soft tissues and skin. Chitosan has a number of advantageous properties, such as biocompatibility, biodegradability, and antimicrobial activity [25,26]. Similarly, it demonstrates immunomodulatory functions by stimulating the activation of epithelial and immune cells. The stimulation of wound healing is seen from the production of proliferative cellular signals, such as interleukins and growth factors, and the activation of corresponding cells [27,28]. All of the abovementioned facts show that biomaterials combined with cellular and acellular compounds form a promising approach to the therapy of the diabetic foot.

This study follows on from our two recently published papers, in which we investigated the effect of a conditioned medium of stem cells and a combination of conditioned medium and chitosan-based biomaterial on wound healing in animal models [29,30]. The present study itself describes the positive impact of autologous stem cells isolated from bone marrow and skin fibroblasts in combination with chitosan-based biomaterial in the therapy of the diabetic foot of a human patient.

## 2. Results

### 2.1. Isolation of MSC from Bone Marrow

**Figure 1:**
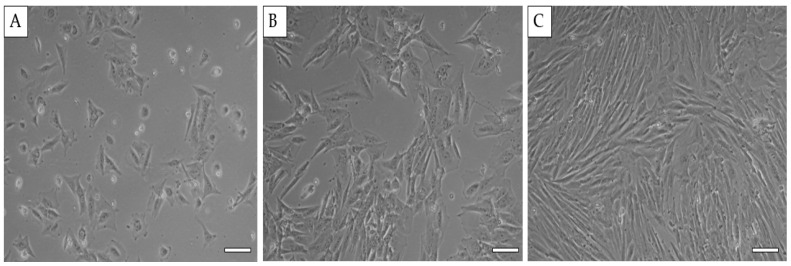
**Morphology of human bone marrow MSC.** BM-MSC passage P0 at day in vitro (DIV) 2 (**A**), DIV9 (**B**), and DIV14 (**C**). Scale bars—50 µm.

Using the below protocol, we were able to isolate and cultivate a homogeneous population of human MSC from bone marrow (hBM-MSC). The yield of isolated cells was 17.9 × 10^6^ cells/mL. Bone marrow MSC showed a fibroblast-like shape, which is typical for MSC (Figure 1).

### 2.2. CD Characterization of Human Bone Marrow MSC

**Figure 2:**
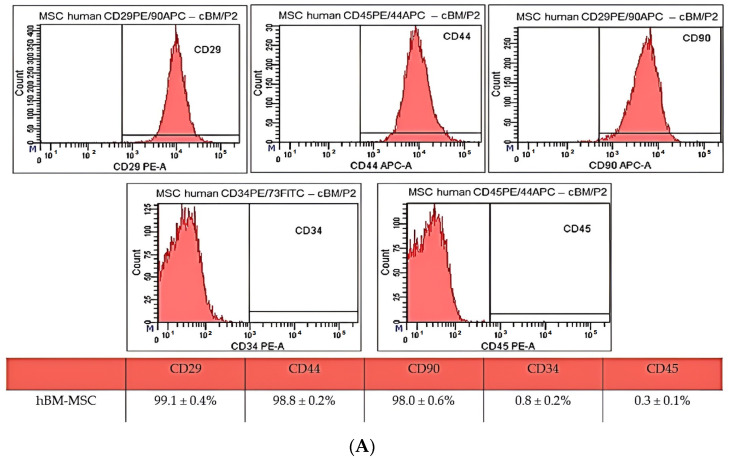

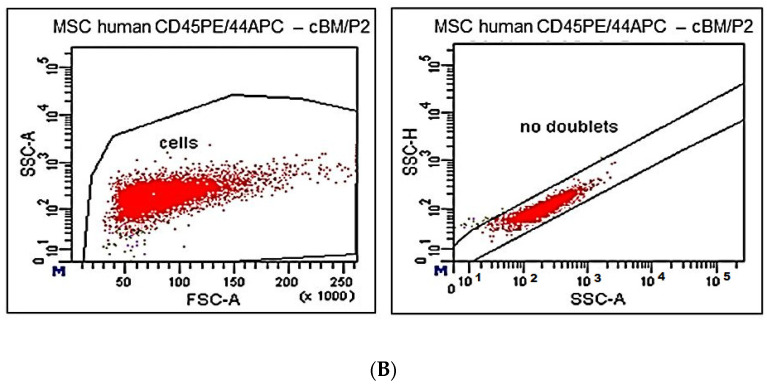
(**A**) **Results of CD analyses of human bone marrow MSC (A) from passage 2 (P2).** hBM-MSC showed high positivity for CD29 (99.1 ± 0.4%), CD44 (99.8 ± 0.2%), and CD90 (98.0 ± 0.6%) and low expression of CD34 (0.8 ± 0.2%) and CD45 (0.3 ± 0.1%). The gating strategy was as follows: forward/sideward scatter and sidewards scatter/sideward scatter pulse height to eliminate debris and doublets. (**B**) **CD analyses of human bone marrow MSC from passage 2 (P2).** The gating strategy was as follows: forward/sideward scatter and sidewards scatter/sideward scatter pulse height to eliminate debris and doublets.

Results of CD analyses of hBM-MSC from passage 2 (P2) (Figure 2A) show high expression of CD29 (99.1 ± 0.4%), CD44 (99.8 ± 0.2%), and CD90 (98.0 ± 0.6%) and low expression of CD34 (0.8 ± 0.2%) and CD45 (0.3 ± 0.1%). We observed a low percentage of autofluorescent cells in hBM-MSC for PE (0.4%) and APC (0.8%). The gating strategy was as follows: forward/sideward scatter and sidewards scatter/sideward scatter pulse height to eliminate debris and doublets (Figure 2B).

### 2.3. Multilineage Potential

**Figure 3:**
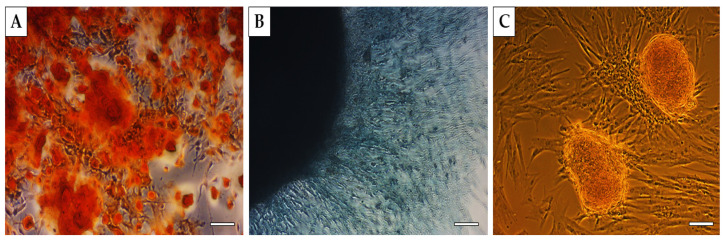
**Multilineage potential of human MSC**. Human bone marrow MSC showed high osteogenic (**A**—presence of calcium deposits detected by Alizarin red) and chondrogenic potential (**B**—presence of glycoproteoglycanes detected by Alcian blue staining); however, isolated cells showed non-specific (low) adipogenic potential (**C**—triglycerides detected by Oil Red O staining). Scale bars—50 µm.

Using a commercial multilineage differentiation kit, recommended culture protocol and staining methods, we confirmed the ability of MSC isolated from human bone marrow to differentiate into osteocytes and chondrocytes and only a non-specific ability to differentiate into adipocytes (Figure 3).

### 2.4. Isolation and Characterization of Human Dermal Fibroblast Primary Culture

**Figure 4:**
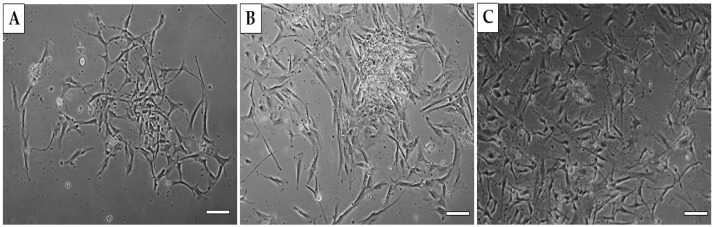
**Morphology of human dermal fibroblasts.** Human fibroblasts from passage P0 at DIV3, (**A**) DIV8, (**B**) and DIV14 (**C**). Scale bars—50 µm.

Using a standard protocol, we were able to isolate and cultivate a homogeneous population of human dermal fibroblasts. The yield of isolated cells was 1.5 × 10^6^ cells/mL. Cells from the cultivated population showed a spindle shape and were 85–95 µm in size (Figure 4).

**Figure 5:**
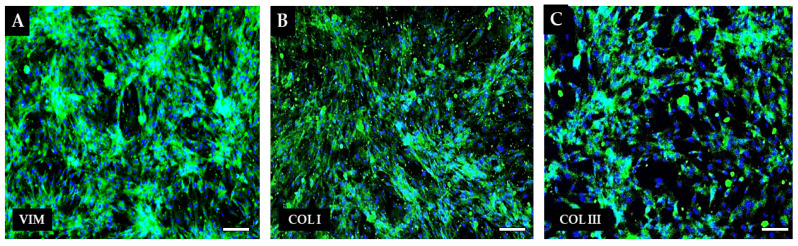
**Immuno-cytochemistry characterization of human dermal fibroblasts.** Expression of vimentin (**A**), collagen I (**B**) and collagen III (**C**) detected by fluorescent microscopy. The nuclei were stained with DAPI. Scale bars—100 µm.

As in our previous publication for immuno-cytochemistry characterization, we used fibroblast markers vimentin, collagen I and collagen III [31,32]. The cultivated population was positive for each of the mentioned markers (Figure 5).

### 2.5. Preparation, Characterization and In Vitro Testing of Biopolymer Scaffolds

The analysis of molecular weight distribution of biopolymers in the PHB/chitosan scaffolds revealed that the average molecular weight (Mw) of PHB and chitosan were 80 and 46 kDa, respectively. The Mw of PHB in the scaffolds was reduced by 30% as compared with the original Mw of PHB, which was around 120 kDa. This is contrary to the small change observed in the Mw of chitosan (Mw of native chitosan is 49 kDa).

**Figure 6:**
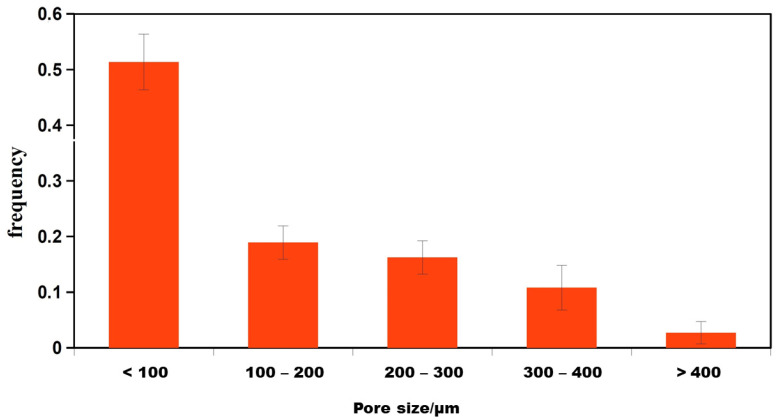
**Pore size distribution determined via image analysis of optical micrographs.** Pore size distribution of PHB/chitosan scaffolds (PCHLY).

The measured open porosity of the PHB/chitosan scaffolds was 90 ± 3%, with a relatively broad pore size distribution as shown in Figure 6.

**Figure 7:**
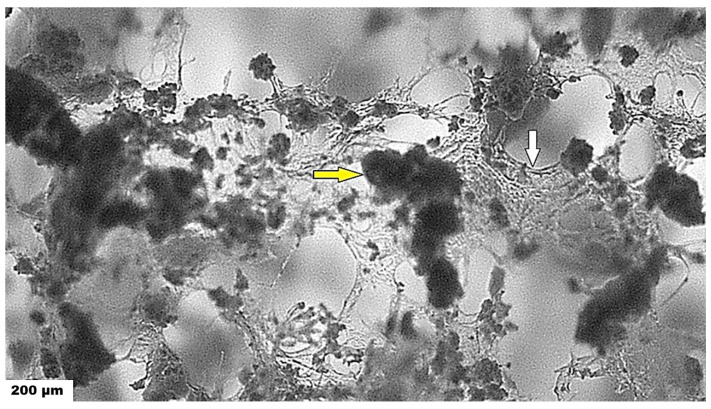
**Microstructure of used scaffold.** Microstructure of scaffold after sterilization (white arrow indicates amorphous gel structures characteristic of chitosan fibers and yellow arrows indicate PHB aggregates of nanoparticles trapped in chitosan net).

The pore size distribution showed a high fraction of <100 µm (around 50%) and a relatively large number of larger macropores (>100 µm), even with the presence of >400 µm pores. This is a good indication of cell motility as well as to the faster diffusion of nutrients to cells and following their proliferation. The macroscopic images of the microstructures of scaffolds obtained from an optical microscope are shown in Figure 7.

In the microstructure of the scaffolds, characteristic plate-like walls composed of transparent chitosan fibers formed a fibrous macroporous network, which is responsible for the formation of spongy-like, effectively trapped, and irregularly shaped coarse aggregates of PHB up to 400 µm in size that are formed by the connection of several globular agglomerates consisting of PHB microparticles. It is clear that the separation of biopolymers results in different surface properties (e.g., surface tension) of the precipitated biopolymers where PHB has a lyophilic nature (enhanced aggregation in aqueous solutions), contrary to chitosan with more hydrophilic character.

**Figure 8:**
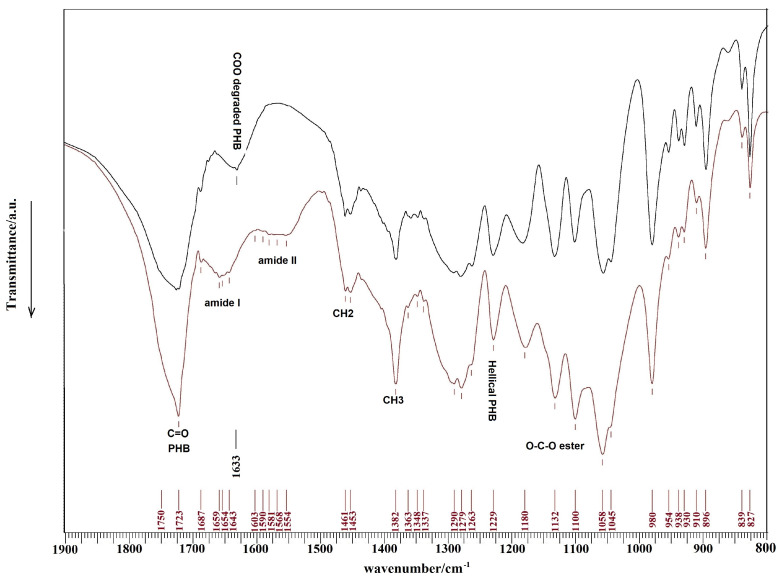
FTIR spectra of PHB standard (black) and PCHLY scaffold (red).

FTIR spectra of PHB standard and PCHLY scaffold are shown in Figure 8. From the comparison of spectra, we can observe that the resultant intensity of PHB peaks is much more intense than those of chitosan and that their mutual overlap caused only change in relative intensities of PHB bands and low intense chitosan amide bands. In the PHB spectrum, very intense PHB bands from stretching vibrations of the C=O group at 1723 cm^−1^, symmetric vibrations of the CH_3_ group at 1382 cm^−1^, scissoring vibrations of the CH_2_ group at 1453 cm^−1^, peaks from asymmetric and symmetric stretching vibrations of the C–O–C ester group at 1180 and 1132 cm^−1^, respectively, and helical conformation of crystalline PHB peak at 1230 cm^−1^ were identified in the spectra. Note that the peak around 1630 cm^−1^ probably originates from the vibration of the carboxylate anion after partial degradation of PHB. In the case of chitosan, bands from C–O stretching vibrations (amide I) at 1650 cm^−1^ and amide II N–H stretching vibrations at 1570 cm^−1^ were found in the PCHLY spectrum. Note that the very weak interaction between the PCHLY biopolymer components may be due to insignificant shifts in the PHB peak maximums (for the extended spectrum of FTIR analysis (800–4000) see the Supplementary Materials Figure S1).

**Figure 9:**
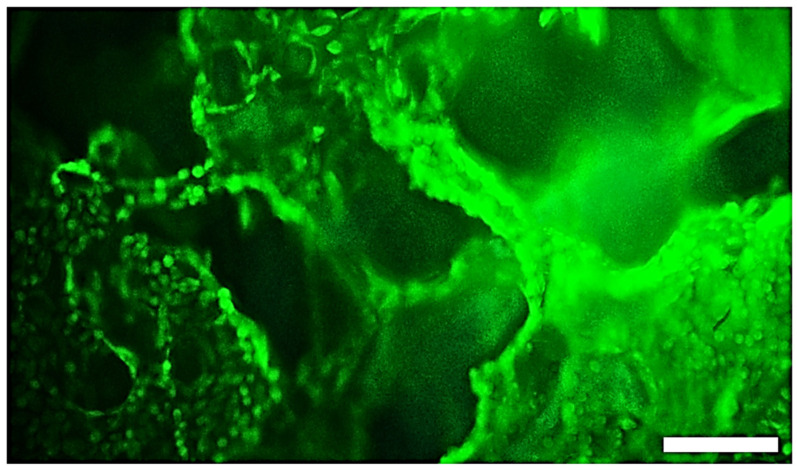
**Non-cytotoxic character of PCHLY scaffold**. Live/dead staining of fibroblasts growth on surface (or pore walls) of PCHLY for 9 days in culture medium (37 °C, 95% humidity and 5% CO_2_). Scale bar 150 µm.

The non-cytotoxic character of the PCHLY scaffolds verified live/dead staining of fibroblast growth on the surface of the substrates and the macropore walls in a dense layer. The micrograph demonstrates the formation of a uniform cell layer on substrates as well as the presence of cells in macropores. This fact indicates the presence of surfaces of biopolymeric walls in pores with open microstructures that are appropriate to allow cell migration into the internal structure of the scaffolds (Figure 9).

### 2.6. Application of Chitosan-Based Biomaterial Combined with Autologous Human Fibroblasts and Bone Marrow-Derived MSC for Treatment of Diabetic Foot Ulcer

**Figure 10:**
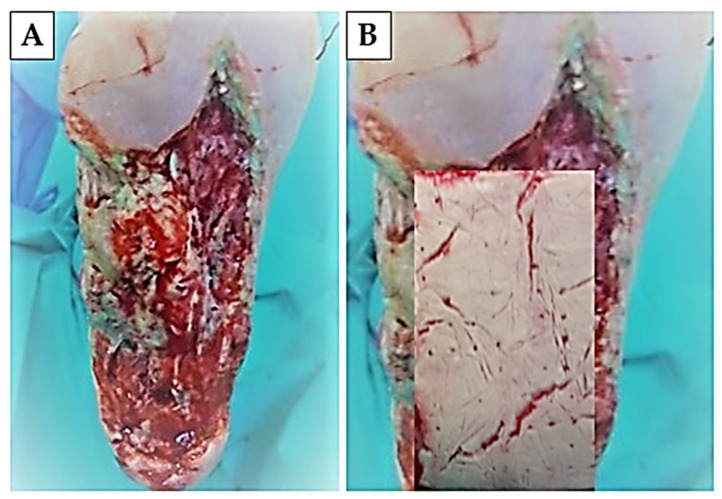
**Treated diabetic foot.** Defect of foot after necrotomy and wound revision (**A**) and defect with applied chitosan-based biomaterial with adhered autologous hBM-MSC and skin fibroblasts (**B**).

**Figure 11:**
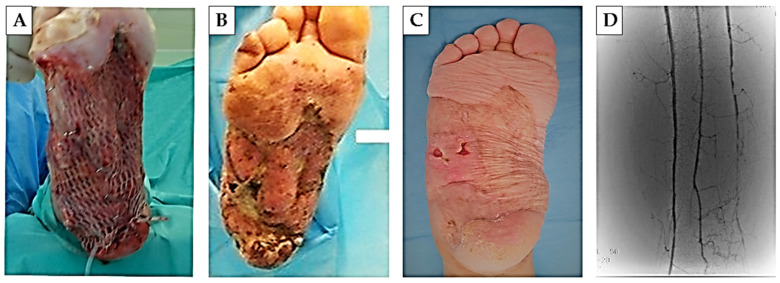
**Wound healing progress.** On the seventh day of treatment, an autologous skin graft measuring 80 cm^2^ was applied (**A**). Skin defect after four (**B**) and eight months (**C**) of therapy. The result of the angio-CT examination after eight months of treatment (**D**).

After the necrotomy and the surgical revision of the foot ulcer, we applied the abovementioned biomaterial with adherent autologous hBM-MSC and dermal fibroblast on the bottom of the revised defect (Figure 10) and applied an autologous hBM-MSC dissociated physiological solution of NaCl, in the form of an intramuscular injection, into the foreleg region. As one of the most important initial findings, we present a significant reduction in pain, 48 h from the start of the treatment, as a subjective feeling of the patient. After seven days of the treatment, we observed no undesirable reaction of the patient’s organism (deterioration of the health condition, breakdown of the internal environment, significant secretion of the wound or formation of pus, CRP decrease from 220 to 45). Due to the extent of the defect, we applied an autologous skin graft taken from the thigh area, which was successfully accepted by the patient’s organism. A period of regular wound dressings and wound care followed (every 72 h), during which we monitored the closure of the defect, the presence of bleeding during the dressings, as well as the formation of granulation tissue. Almost complete healing of the ulcer occurred eight months after the start of therapy. The success of the treatment is also demonstrated by the result of the angio-CT examination, when we observed the presence of blood vessels (Figure 11).

## 3. Discussion

The main goal of this study was to observe the effect of the local and intramuscular application of autologous hBM-MSC and skin fibroblasts with a combination of chitosan-based biomaterial in the treatment of a diabetic foot after the previous failure of conventional therapy.

For the isolation of autologous hBM-MSC harvested from iliac crest we used a simple method of centrifugation and size separation using a cell strainer (100 µm). The yield of hBM-MSC isolated using this method was 17.9 × 10^6^ nucleated cells/mL, which is closely correlated with other studies that describe a yield of isolation between 5.8 and 38.7 × 10^6^ nucleated cells per mL of suspension [33,34]. Isolated hBM-MSC show a fibroblastoid-like shape and size of 110–160 μm. The expression of CD surface markers in MSC populations was tested using flow cytometry. During the analysis, we found consistent high expression of CD29, CD44 and CD90 (≥90%) and almost no cells expressed CD34 and CD45 in 2nd passage. These results correlate with the subsequent multilineage potential assay, where we describe only poor or non-specific adipogenic potential, because the high percentage of CD29 and CD90 positive cells in the population reduces adipogenic activity [35]. Next, a high expression of CD29 and CD44 can be seen in the above data to be superior to strong chondrogenic potential [36]. For our experiment, the expression of CD90/Thy-1 played an important role, because it is implicated in angiogenesis and blood perfusion during wound closure [37]. The results of the analysis of multilineage differentiation potential prove that the isolated population of hBM-MSC has a significant osteogenic and chondrogenic potential. However, as mentioned, we confirmed only poor and non-typical adipogenic potential, even when repeating the experiment and after extending the cultivation time in the adipogenic culture medium. The same facts have been described in other studies [38,39]. These circumstances could be explained by the addition of osteogenesis-mediating and adipogenesis-suppressing bioactive molecules, such as Runx2, Wnt10b ALP, OSX and RhoA, or adipogenesis-enhancing bioactive molecules suppressing osteogenesis, including PPARγ, P2X6, LIF, sFRP-1 and BMPs, which also play an important role [38,40,41,42]. Thus, differentiation to mesoderm cell lines (osteocytes, chondrocytes, fibrocytes, and myoblasts) is the key property required for hard and soft tissue regeneration in veterinary and human medicine [43,44]. Concluding from the abovementioned facts, bone marrow represents the richest, most used and most researched source of stem cells for use in regenerative medicine, despite the relative difficulty of obtaining the source tissue for isolation [45]. MSCs play an important role in the wound-healing process. They participate in the homeostasis phase by promotion of coagulation due to the high tissue factor content and phosphatidylserine [21]. MSCs modify the inflammatory phase by the production of bioactive molecules that polarize M1 pro-inflammatory macrophages to the M2 anti-inflammatory type or suppress the effect of NK cells via their capacity to downregulate TNF-α-dependent inflammation and by triggering TGF-β1 production, which is a key role in wound healing and inflammation control [21,46]. These statements also correlate with our results, as the inflammatory values of CRP decreased from 220 to 45. Similarly, the results concerning the decrease in serum CRP values after the application of MSC have also been observed in recent studies [47,48,49].

Mesenchymal stem cells can be applied to the affected area locally through scaffolds and carriers or systemically by intravenous or intramuscular injection. In our study, we successfully used a combined method of application, where autologous hBM-MSCs were applied both in the form of an intramuscular injection into the foreleg of the affected limb and at the same time locally in the form of a chitosan-based scaffold with adhered hBM-MSC and fibroblasts. Autologous fibroblasts were isolated from a skin sample via enzymatic methods and characterized by immunocytochemistry method using specific antibodies—vimentin, collagen I and collagen III—which are widely respected markers for fibroblasts [50,51]. These orchestrate the whole repair process by producing a number of regulatory molecules, such as TNF-α, INF-γ, and interleukin 1, 6 and 12; chemokines CXCL1, CX3CL1 or CCL2; and crosstalk with the other cell populations involved in the healing mechanisms [52,53]. Other authors have also used a similar procedure in their studies [54,55,56]. The therapeutic effect can be attributed to all of the components—cellular as well as chitosan-based biomaterial. Chitosan, as a natural biodegradable material, is present at all stages of wound healing. It affects the hemostatistic phase by inducing platelet and plasma protein aggregation, promoting coagulation and vasoconstriction at the site of injury [57]. It also potentiates the activity of polymorphonuclear leukocytes, macrophages and fibroblasts, which shortens the inflammatory phase and accelerates the onset of the proliferation phase [58]. In the proliferative phase of healing, it stimulates the secretion of cytokines such as transforming growth factor-β (TGF-β), platelet-derived growth factor (PDGF), interleukin-1 (IL-1) and interleukin-8 (IL-8) [26,59]. It also indirectly affects angiogenesis, dermis repair and epidermis regeneration [60]. In the remodeling phase of healing, it helps the formation of 3D structure and the formation of collagen fibers [27].

The combination of chitosan-based biomaterials and a cellular component represents a promising approach in DFU therapy, as evidenced by partially published studies. Escárcega-Galaz et al. have proved the positive effect of chitosan in the form of gel or film in the treatment of diabetic wounds in eight patients [61]. Velazcoet al. have described the healing of DFU in a 48-year-old patient as early as the 45th day of treatment, using a chitosan-based biomaterial [62]. Considering the scope of the defect in our case, it is not possible to compare this period of treatment. Our patient described significant pain relief as soon as 48 h after local and intramuscular application of stem cells. This phenomenon can be described from several points of view. The first is the already mentioned immunomodulating potential and the production of chemokines and trophic factors. Another is the influence of signaling pathways directly related to pain, namely the inhibition of the Wnt/β-catenin signaling pathway [63,64].

In view of the lack of presented clinical results concerning the treatment of DFU using chitosan and mesenchymal stem cells or fibroblasts from the field of human medicine, it is necessary to approach each patient individually and carefully consider the treatment procedure.

## 4. Materials and Methods

First, we must obtain the informed consent of a patient. This is the essential criterium when seeking approval of the study from the Ethical Committee of Agel Hospital in Košice-Šaca. The study was approved by the EC of Agel Hospital in Košice-Šaca on 13 September 2023 (15-2023).

### 4.1. Isolation of MSC from Bone Marrow

Bone marrow for the isolation of autologous MSC were obtained during surgery practice under strictly sterile conditions from crista illiaca of os illium under general anesthesia, using the standard Jamshidi™ Bone Marrow Biopsy Needles (16 ga). Donor—female, 39 years, 80 kg. Aspirated bone marrow (120 mL) was diluted in sterile phosphate-buffered saline solution supplemented with antibiotic (PBS; Gibco, Basel, Switzerland) and centrifuged at 500× *g* for 10 min. The obtained pellet was resuspended in Dulbecco’s Modified Eagle Medium/Nutrient Mixture F12 (DMEM-F12) culture medium + 10% Fetal Bovine Serum (FBS) + 2% ATB-ATM (all Biowest, Nuaillé, France). Cells were plated in a T75 culture flask at a concentration of 10^6^ cells/mL and incubated in culture medium (DMEM-F12) + 10% FBS + 2% ATB + ATM at 37 °C and 5% CO_2_. Non-adherent cells were removed, and the medium was subsequently changed twice a week.

### 4.2. Cell Passaging Procedures

When the cultivated cell population reached a confluence of approximately 85–90%, we proceeded to cell passaging. To separate the cells from the surface of the culture flask, an enzymatic trypsinization method using trypsin–EDTA 1× (Biowest, France), which acted on the cells depending on the level of confluence, was used for 5–7 min at 37 °C. To inactivate the trypsin, FBS in a 1:1 ratio was used and the whole suspension was subsequently centrifuged at 400× *g* for 10 min. The supernatant was removed, and the cell population was plated on T75 culture flasks at a concentration of 1.5 × 10^6^ cells/flask.

### 4.3. CD Characterization of Bone Marrow MSC

For the flow cytometry procedure, bone marrow MSCs from passage 2 (P2) were used. Samples were analyzed for CD29 and CD44, CD90-positive and CD45- and CD34-negative cells. Each sample was diluted to a final concentration of 2 × 10^5^ cells and centrifuged at 400× *g* for 5 min. Subsequently, the supernatant was removed and the cell pellet was resuspended in 100 μL of PBS containing 3–5 μL of CD90 (MA5-16671, monoclonal antibody, allophycocyanin (APC)), CD29 (12-0299-42, monoclonal antibody, phycoerythrin (PE)), CD44 (17-0441-82, monoclonal antibody, APC), CD34 (CD34-581-04, monoclonal antibody, PE) and CD45 ((12-0459-42, monoclonal antibody, PE) (all Invitrogen, Carlsbad, CA, USA)) and incubated for 60 min at 4 °C in the dark. At the end of the incubation period, the samples were centrifuged again at 400× *g* for 5 min, the supernatant was removed, and the sample was washed in 200–500 µL of washing solution (1% BOFES in PBS + 0.1% sodium azide). Cytometric analysis was performed on a BD FACSCanto^®^ flow cytometer (Becton Dickinson Biosciences, Franklin Lakes, NJ, USA) equipped with a blue (488 nm) and a red (633 nm) laser and 6 fluorescence detectors. The percentage of cells expressing individual CD markers was determined by a histogram for the respective fluorescence. The data obtained via measurement were analyzed in BD FACS Diva^™^ analysis software (version 8.0). As a negative control, the same type of non-marked MSC for the control of autoflorescence was used. The gating strategy for flow cytometry was performed by forward/sideward scatter and sidewards scatter/sideward scatter pulse height to eliminate debris and doublets. The viability of observed cells varied between 85 and 96%.

### 4.4. Multilineage Potential

To confirm the multiline potential of the BM-MSC, the MesenCult ACF Chondrogenic Differentiation Kit, MesenCult Osteogenic Stimulatory Kit and MesenCult Adipogenic Differentiation Kit (STEMCELL Technologies, Vancouver, BC, Canada) were used according to the manufacturer’s instructions. The cells used for multiline differentiation were from P2. Cells were cultured in 24-well plates with an initial density of 5 × 10^4^ cells/well for osteocytes, 5 × 10^4^ cells/well for adipocytes, and 6 × 10^4^ cells/well for chondrocytes. Each micromass represented a single drop of 5 μL 6 × 10^4^ cells, which was placed in the center of the well and then incubated at 37 °C and 5% CO_2_ for 2 h for better adherence to the surface. Then, 500 μL of chondrogenic medium was added. After the recommended culture time (21 days), the cells were fixed using 4% paraformaldehyde (PFA), and the individual populations were stained with the Alizarin red staining method for evidence of calcium deposits in the osteoblast population, Alcian blue for the detection of proteoglycans in the chondroblast population, and Oil red (all Sigma Aldrich, Saint Louis, MO, USA) for the staining of fat vacuoles in the adipocyte population.

### 4.5. Isolation of Dermal Fibroblasts

Autologous human fibroblasts were isolated from skin samples (2 × 1 cm) obtained during previous surgery practice (bone marrow collection) under strictly sterile conditions from the place of the incision. After harvesting, samples were transported in DMEM medium (Biowest, France) with 2% ATB-ATM in sterile conditions. Skin samples were then washed in 70% ethanol solution and twice in PBS. In the next step, samples were cut into small pieces (0.5 × 0.5 cm). To divide the dermis and epidermis layer, an enzymatic method of dispase II (Gibco, Basel, Switzerland) digestion at a concentration of 2.0 U/mL at 37 °C for 1 h was used. After dispase digestion, the dermis was mechanically separated from the epidermis. For the isolation of fibroblasts from the dermis, enzymatic digestion by collagenase IV (Gibco) at a concentration of 0.05 mg/mL for 6 h at 37 °C was used. The cell suspension was centrifuged for 8 min at 400× *g*. Cells were plated in 6-well plates (3 × 10^5^ cells/well) and cultured in DMEM-F12 supplemented with 10% FBS, 2% ATB-ATM, basic fibroblast growth factor (bFGF) (20 ng/mL, Milipore, Burlington, MA, USA), and epidermal growth factor (EGF) (20 ng/mL, AppliChem, Darmstadt, Germany) at 37 °C in 5% CO_2_ incubator for 4 days in vitro (DIV4).

### 4.6. Immunocytochemistry Characterization of Canine Skin Fibroblasts

Human dermal fibroblasts (DIV4) were fixed with 4% PFA for 15 min and incubated with anti-vimentin primary antibody (mouse monoclonal, Invitrogen; MA5-11883; 1:200), anti-collagen I (rabbit polyclonal; Invitorgen; PA1-26204, 1:200) and anti-collagen III (mouse monoclonal; Invitrogen; CSI 007-01-02, 1:200). Cells were incubated with primary antibodies in PBS with Triton X-100 (0.1%, Thermo Fisher Scientific, Waltham, MA, USA) overnight. Secondary antibodies conjugated with FITC (green goat anti-mouse, Invitrogen, 62-6511, 1:500 and goat anti-rabbit, Invitrogen, F-2765, 1:500) were used. The nuclei were stained with 4, 6-diamidino-2-phenylindole (DAPI, Sigma Aldrich, St. Louis, MO, USA). Incubation time for secondary antibody and DAPI was 1 h. The staining was detected by fluorescent microscopy (Zeiss, Oberkochen, Germany) and pictures were taken with a fluorescent microscope camera (Zeiss Axiocam ERc 5s, Zeiss, Germany).

### 4.7. Preparation of Biopolymer Scaffolds

**Figure 12:**
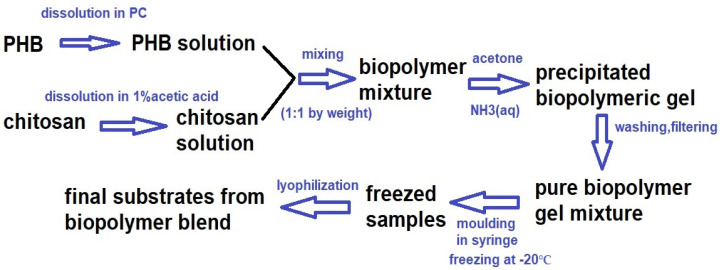
Scheme for biopolymer blend preparation.

Polyhydroxybutyrate (GoodFellow, Roy, UT, USA)/chitosan blends (Sigma Aldrich, low molecular weight) were prepared by mixing PHB (dissolved in propylene carbonate (at 130 °C), 2% (*w*/*v*) solution) and chitosan solutions (in 1% acetic acid, 1% (*w*/*v*) solution) using a magnetic stirrer at 400 rpm after the addition of 1 mL of NH_3_ (aq, 25%, Fluka, Buchs, Switzerland) and acetone (Sigma Aldrich, for analysis). The weight ratio of biopolymers was equal to 1:1. The final blends were filtered, washed with distilled water and loaded to a polypropylene syringe (50 mL volume) to form disc-shaped samples (4 cm in diameter and 5 mm in height) and frozen at −20 °C. The scaffolds were obtained by lyophilization (Ilshin lyophilizer, iLShin Biobase, Ede, The Netherland) for 8 h. The final biopolymer scaffolds were sterilized in an autoclave at 121 °C (Figure 12).

The water uptake was determined by soaking the scaffolds (approx. 100 mg) in 0.9% NaCl solution at 37 °C until a constant mass of samples. Soaking was done three times, and the water uptake was calculated as the ratio of the weight of the wet sample to the original dry sample. The calculated value was equal to 5.2.

The open porosity of the PHB/chitosan scaffolds was measured by filling the pores with ethanol (Sigma Aldrich, absolute, HPLC grade) after exhausting the air from scaffolds. Porosity was calculated as the ratio of the volume of ethanol in the pores (obtained from the difference between the mass of wet and dry sample and the density of 100% ethanol) and the volume of dry sample measured from sample dimensions.

#### 4.7.1. Characterization of Porosity, Average Molecular Weight of Biopolymers in Scaffold, FTIR Analysis of Composites

The microstructure of scaffolds was observed with an inverted optical fluorescence microscope (Leica DM IL LED, Leica, Wetzlar, Germany) equipped with a CCD camera in VIS mode at 50× magnification. The pore size distribution was evaluated from 2 mm^2^ of surface area (five different locations) by image analysis (Image J8 software). Pore sizes were divided into five pore size fractions.

The average molecular weight (Mw) of chitosan and PHB was measured by gel permeation chromatography (GPC, Watrex, Prague, Czech Republic). Chitosan separation was undertaken on a PL gel mixed OH 8 μm column at a mobile phase flow rate of 1 mL/min (mobile phase 0.1 M NaH_2_PO_4_, pH = 6) using a UVVIS detector (SYKAM S3240, Eresing, Germany) at 230 nm. PHB separation was performed on a PL gel mixed C 5 mm column with chloroform as the mobile phase and an RI detector (Schambeck SFD, RI 2000, North Rhine-Westphalia, Germany). The average molecular weights of chitosan and PHB were determined from calibration curves using dextran (chitosan) and polystyrene (PHB) as standards with different average molecular weights and calculated by Clarity DataStation software (version 4.0.4).

Mutual interaction of biopolymers was analyzed using FTIR spectroscopy (Shimadzu, IRAffinity1, KBr method, Kyoto, Japan).

#### 4.7.2. In Vitro Cytotoxicity Testing

The L 929 mouse fibroblasts (ECACC, Salisbury, UK) were used for cytotoxicity testing. Cells were cultured in culture flasks with surface areas of 75 cm^2^ in minimum essential medium Eagles (MEM) with 10% fetal bovine serum and ATB-antimycotic (penicillin, streptomycin, amphotericin) solution (all Sigma Aldrich, MO, USA) at 37 °C in 5% CO_2_ atmosphere and 95% humidity in an incubator (Memmert, Schwabach, Germany). The medium was changed every 2 days. After the cells reached about 80% confluence, they were harvested by trypsinization using 0.25% trypsin–EDTA (Sigma Aldrich, MO, USA) solution followed by the addition of fresh medium to create a cell suspension. The cell density in suspension was calculated using a Neubauer hemacytometer.

The samples for in vitro testing were cut with a corkscrew from prepared sterilized scaffolds. The sterile samples (Ø6 mm, height 2 mm) were put in the wells of a 48-well cell culture plate (Sarstedt, Nümbrecht, Germany) and seeded with 2.0 × 10^4^ cells in 500 μL (4.0 × 10^4^ cells/mL) of complete medium and cultured at 37 °C in 5 vol % CO_2_ and 95% humidity in an incubator. The morphology, density and distribution of cells on polymeric samples was determined by live/dead cell staining using fluorescein diacetate/proidium iodide dye. The cells on substrates were observed by a fluorescence inverted microscope (Leica DM IL LED, blue filter) after 9 days of cultivation.

### 4.8. Application of Chitosan-Based Biomaterial Combined with Autologous Human Fibroblasts and Bone Marrow-Derived MSC for Treatment of Diabetic Foot Ulcer

Patient was a woman, 39 years old and 80 kg). Their medical history indicated the long-term treatment of type I diabetes mellitus, as well as thyroid disease and high blood pressure. The patient was admitted to the Department of Burns and Reconstructive Surgery of the Agel Hospital Košice-Šaca with an extensive foot ulcer of the right pelvic limb due to the indication of the need for amputation of part of the limb. Clinical examination showed necrotic changes of the foot, lytic changes of Lisfrank, dislocation in II.–V., and subluxation of I. Stp. Fr. II. and III. MTT.

**Figure 13:**
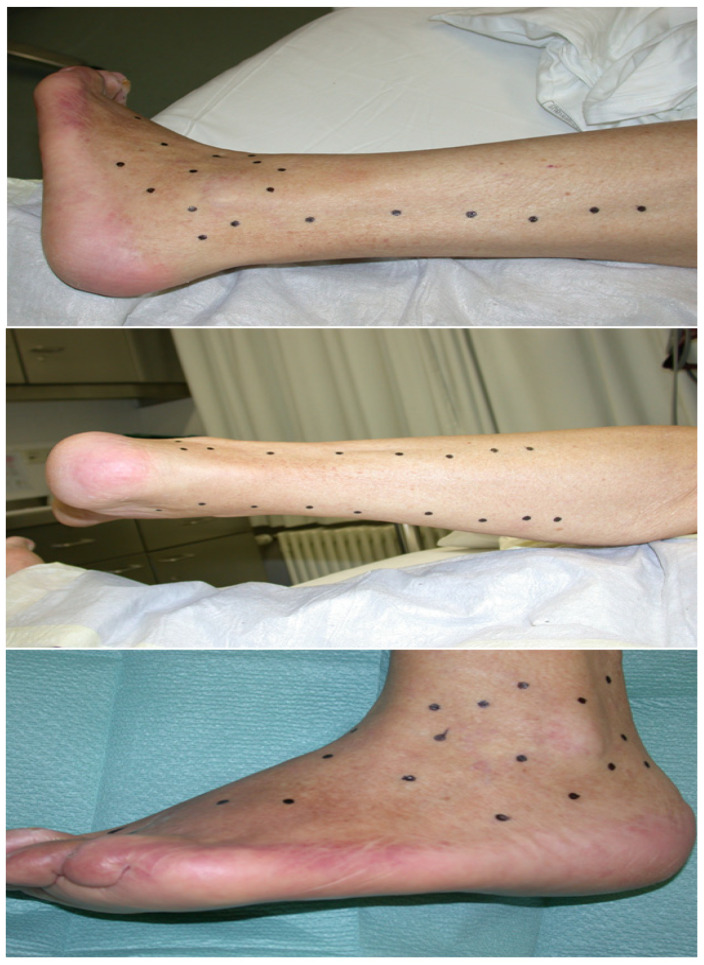
Scheme for intramuscular application of autologous hBM-MSC. An amount of 0.1 mL of cell suspension was applied to each highlighted point.

In the supine position, after preparation of the operative field, we performed a tangential necrotomy in the area of the planta and calcaneus of the right foot. Due to the previous failure of conventional treatment, we approached the application of chitosan-based biomaterial with adhered autologous BM-MSC and skin fibroblasts (8 × 10^4^ BM-MSC/cm^2^ and 15 × 10^3^ fibroblasts/cm^2^) as well as the local intramuscular application of autologous BM-MSC to the foreleg area (2 × 10^6^ cells/10 mL of 0.9%NaCl) (Figure 13). Cells of both lines for application came from the second passage. Adherence to the biomaterial was performed under sterile tissue laboratory conditions. For the adhesion itself, we applied BM-MSC in a concentration of 80,000 cells/cm^2^ and 15,000 fibroblasts/cm^2^, which were applied to the wound surface of the biomaterial and adhered for 2 h under standard conditions (37 °C and 5% CO_2_). The secondary layer of the outer covering was a sterile gauze bandage (Hartmann, Heidenheim an der Brenz, Germany) soaked in suspensio visnievski cum balsamo peruviano (Fagron, KE Capelle aan den IJssel, The Netherlands) and the tertiary layer of the outer covering was synthetic cotton wool (Cellona, Lohman and Raucher Int., Rengsdorf, Germany). Re-bandaging of the second and tertiary layers was performed at 72 h intervals, taking into account the condition and progress of wound secernation. As a support therapy, the patient was given a combination of NSAIDs ((meloxicam (0.2 mg/kg, meloxicam, Generics Ltd., Hertfordshire, UK) + metamizole (15 mg/kg, Novalgin, Sanofi Aventis, Mumbai, India)) and convenient antibiotic therapy, according to the results of bacteriological cultivation (Ceftriaxon, 1 × 1 g i.v., Sandoz, Basel, Switzerland). Due to the extent of the defect, on the seventh day from the beginning of the therapy, we proceeded to remove the autograft (dermal graft) from the thigh area. The procedure was performed under general anesthesia, the thickness of the graft was 0.254 mm, and the size was 80 cm^2^ and it was fixed by suture. The primary layer of the outer covering was a sterile gauze bandage (Hartmann, Germany) soaked in suspensio visnievski cum balsamo peruviano (Fagron, The Netherlands) and the secondary layer of the outer covering was synthetic cotton wool (Cellona, Lohman and Raucher Int., Germany). Re-bandaging of the second and tertiary layers was performed at 72 h intervals, taking into account the condition and progress of wound secernation.

## 5. Conclusions

The presented pilot study describes the positive impact of autologous bone marrow-derived mesenchymal stem and dermal fibroblast in combination with chitosan-based biomaterial in diabetic foot treatment.

- In the present study, we successfully isolated mesenchymal stem cells from the bone marrow. The yield of isolated cells was 17.9 × 10^6^ cells/mL. hBM-MSC showed a high expression of CD29 (99.1 ± 0.4%), CD44 (99.8 ± 0.2%), CD90 (98.0 ± 0.6%) and low expression of CD34 (0.8 ± 0.2%) and CD45 (0.3 ± 0.1%).
- We were also able to isolate and cultivate a cell culture of dermal fibroblasts. The yield of isolated cells was 1.5 × 10^6^ cells/mL. Cells from the cultivated population showed a spindle shape and 85–95 µm in size and, according to the results of immunocytochemistry staining, showed positivity for vimentin, collagen I and collagen III.
- We observed pain relief after the intramuscular application of autologous hBM-MSC, enhancement of healing process activation, formation of blood vessels and almost complete healing of the defect after skin graft transplantation and 8 months of therapy.
- The success of the treatment in this case can be explained by the synergistic positive healing effect of both the used material and the cell components, as fibroblasts and MSC show significant immunomodulating and paracrine potential, which plays an important role in the wound-healing process. In the same way, chitosan-based biomaterials are also used in the healing process of refractory wounds, which directly affects the hemostatic, proliferative and immunomodulating phase [52,54,57].

## Data Availability

The data presented in this study are available upon request from the corresponding author.

## Supplementary Materials

The supporting information can be downloaded at: https://www.mdpi.com/article/10.3390/ijms25158388/s1.
