# Supplementary material for: The Combination of Chitosan-Based Biomaterial and Cellular Therapy for Successful Treatment of Diabetic Foot—Pilot Study
Source: Int J Mol Sci. 2024 Aug 1;25(15):8388. doi: 10.3390/ijms25158388 (PMC11313444; doi:10.3390/ijms25158388)
Supplement: Supplementary file 1 [file ijms-25-08388-s001.zip › ijms-3102373-supplementary.pdf]

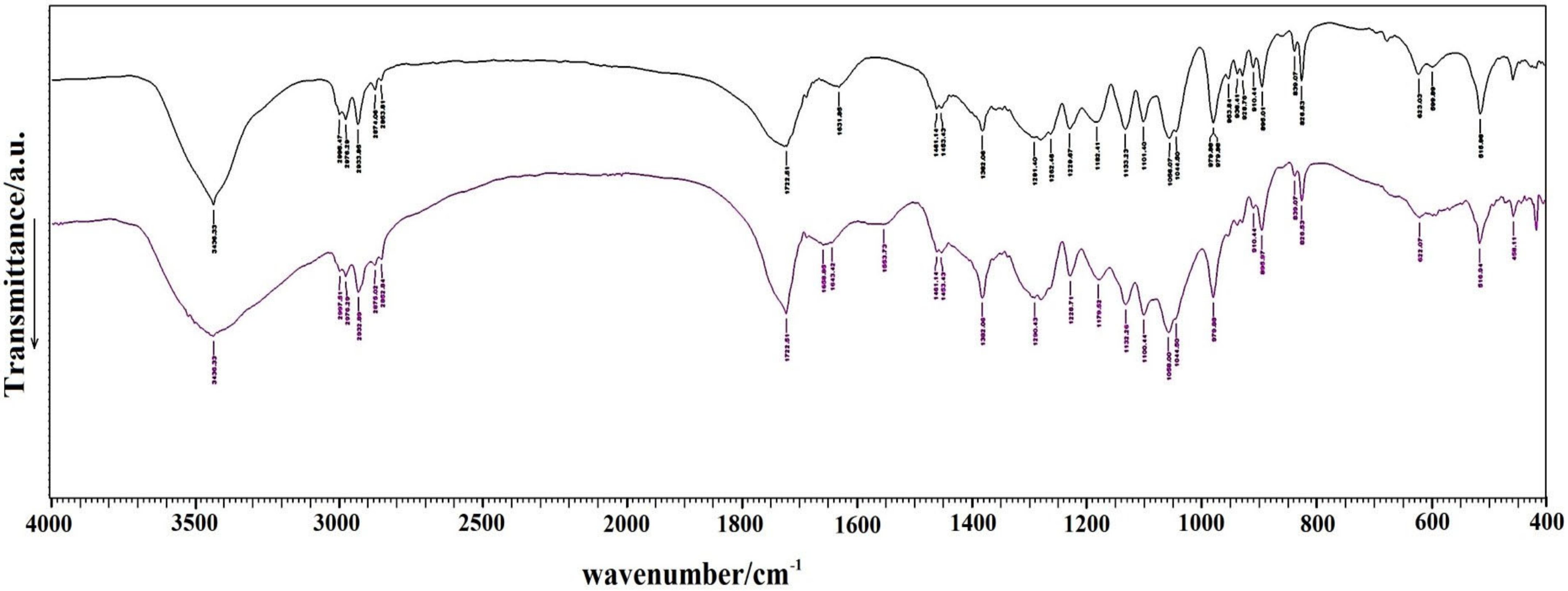

Figure S1. FTIR spectra of PHB standard (black) and PCHLY scaffold (purple). Figure shows extended analysis - range 800 – 4000 of PCHLY scaffold (following Figure 8 in the main text). Only the vibrations of the CH_3, CH_2 groups (all around 2800-2900) and the end OH group in PHB (3437) are visible, which are so few that they are insignificant as the previous vibrations. Chitosans +OH and NH_2 groups (practically invisible except for the broadening of the band from 3500-3200).
